# Getting into Practice

**Published:** 1889-09-14

**Authors:** 


### Getting into Practice.

The question of questions with hundreds of young medical men who are fully fledged as to degrees and hospital experience is : How and where to begin practice. A year ago, or it may be two or three years ago, or even more, the youthful practitioner's one anxiety was "to get through." He used to think that as soon as he could write M.B., or M.R.C.S., after his name his troubles would be over. He has since found out, however, that though it may be a difficult thing to get a qualification it is much more difficult still to get a practice. Unless a man selects one of the services, or "goes in for a specialty," the problem for him resolves itself into a two-horned difficulty. "To buy or not to buy : that is the question." Whether 'tis better to pay down one, two, or three thousand pounds for an introduction to a good partnership, or to keep the money safe in the banker's hands, and putting up a brass plate *de novo*, to trust one's frail bark upon the sea of fortune. The double-barrelled question is easily asked ; to answer it is by no means so easy.

Some men may begin in any place and in any way they choose ; they are sure to command success. But those are few. The majority, however, can please themselves whether they "buy" or "put up their brass plates" without buying, provided they have patience to wait and resources enough to keep them going for a period varying from four to ten years. A good many should certainly buy practices ; and not a few should make sure that they are "unopposed." In all so-called professions there are a considerable number whose first consideration is a "position" in which they will be recognised as "gentlemen." They have no particular liking for one kind of work more than for another. If they could have a choice, it would be that they should do no work at all ; but since their ancestors have not provided them with sufficient means to lead lazy lives, they prefer the certainty of a professional position to the more equivocal status and responsibilities of a man of business. Such men as these always get through life with a minimum expenditure of all kinds of energy. They hate work, and they hate worry ; and provided the cupboard is full, the back clothed, and the house furnished with sufficient comfort and respectability they are content. Men of this class should never by any chance attempt anything but "unopposed" practices. In such practices they are comparatively safe ; for he that has no rival must of necessity be king.

But the ordinary medical graduate is made of better stuff than that. It may fairly be presumed that his mental capacity is above the average, and that his industry up to the present time has been considerable. If not, how has he managed to possess himself of any kind of license to practise? It is, moreover, certain that the character of the ordinary medical graduate is good, much better, indeed, than that of the ordinary medical student. If it be asked how can the graduates be better than the students of medicine, seeing that they are drawn exclusively from the student class, the answer is both easy and convincing. It is almost always the vicious student who is the lazy student, and he never becomes a graduate at all. On the other hand, the student who works hard is usually also self-respecting and of good conduct, and it is from his ranks that the actual practitioners of medicine are largely recruited. Even if there had been no such obvious explanation as this, or no explanation at all, the fact itself that the young practitioner of medicine is nowadays for the most part a young man of good principles and wholesome character is very evident. Such a man, under favourable circumstances, will develop into a good husband and father, and a worthy citizen.

But the young practitioner, though a pleasant and good fellow, is almost always entirely destitute of business experience and knowledge of the world. On the other hand, he is often extremely conceited. The knowledge of a smattering of many sciences of which even the more educated of his fellow-men are almost entirely ignorant, and the power of putting the magic letters M.B. or M.R.C.S. after his name, make him think himself very superior indeed to ordinary mortals. If, in addition, he has the good fortune—or shall we say the misfortune?—to be an honours man, he is apt to carry his head so high that no ordinary doorway will admit him at all. There is, undoubtedly, a childish, though, perhaps, not an unamiable crudeness about youthful medicos which is decidedly amusing to experienced men of the world ; but which often leads the young men themselves into encounters and rebuffs that are by no means pleasant. If every young doctor would remember that a degree, however good it may be, is not a practice, but only an instrument for enabling him to acquire one ; not a battle, but only a sword by means of which, in capable hands, a battle may be won, he would be saved many a heartache. The best degree in the world, like the best sword, will never conduct an incapable, or a worthless, or an idle, or a stupid fellow to victory. The degree is but the very first step. The ladder of fame, of useful service, or of financial success, is both long and steep, and may, nay, must, in almost every case, take many years to scale. A good degree, like an inherited fortune or a famous name, may be the ruin of a man, and will be so if he thinks that by it alone he is going to carve his way to success. Of course, in the hands of a young man of sense and modesty, a good degree is an excellent recommendation.

All these facts being duly considered, the question still remains for the average young doctor, What is the best way of getting into practice? From a personal experience of different ways, and from anxious consideration of many other cases than his own, the present writer gives the preference, without any hesitation, to a good partnership. A good partnership has many advantages, and some disadvantages ; but the advantages outweigh the disadvantages altogether. To begin with, a man settles down to earnest and responsible work at once in a good partnership. He thus begins in the most rapid and thorough way to acquire medical experience. For, however valuable the experience gained as a house surgeon and house physician may be, it can never produce the same effect on mind and character as work for which a man himself bears the sole and entire responsibility, and which he feels may lead him to the most solid and brilliant success, or to the profoundest failure and disgrace. On the other hand, the man who "puts up his doorplate" without buying a partnership or a practice, may by chance have a little work to do the first year or two, and a little more in his third and fourth years. But the practical certainty is this, that the man who enters upon a good partnership will have seen more good work, and acquired more varied and solid experience, in one year, than the man who begins without buying will have done and acquired in four or five years. That is a result which can hardly be bought too dear. Medical men are apt, very naturally, to look principally, or almost exclusively, at the financial results of purchasing partnerships or otherwise. There can be no greater mistake. Practical experience, and the trained skill which results from it, are of priceless value. Many other considerations might be urged in favour of suitable partnerships, and no doubt something might be said against them. Space does not permit of a long argument. One other circumstance is well worth mentioning. When a man enters upon full work, he enters upon what is pretty sure to make him cheerful and happy. When he enters upon a doubtful period of waiting he enters upon a period of wearing anxiety, which may spoil his temper, make his sleep less sweet, impair his health, and injure his character. Very few men can "wait" for success so happily and contentedly as they can "work" for it. Endurance is much more trying than work, and demands far higher qualities of mind and soul. The average man will do well not to risk it unless he is compelled.

In any case "getting into practice" is a decidedly serious step ; a step charged with more practical and life-long issues than any the young graduate has yet taken, unless he has been foolish enough to marry. A man must be emphatically a fool who does not give to its consideration the best brains he has ; and if he be wise he will search out one or more of his medical acquaintances whose character and good sense he can rely upon, and who have reached the mature age of forty. "At thirty a man suspects himself a fool, knows it at forty, and reforms his plan." When a man is old enough to have found out the possibility of his being a fool himself, he has reached the age when, with becoming modesty, he may venture to offer advice to others.

